# Forewarned but not forearmed: The risk of science driven by immediacy

**DOI:** 10.1017/ice.2020.350

**Published:** 2020-07-22

**Authors:** Daniel Moreno Soto, Walter D. Cardona Maya

**Affiliations:** Reproduction Group, School of Medicine, University of Antioquia, Medellín, Colombia

*To the Editor—*The coronavirus disease 2019 (COVID-19) pandemic has killed hundreds of thousands, has infected millions, and has ravaged the economy worldwide in <6 months. The full implications of this crisis are still unfathomable and might span years or even decades. The virus has bewildered everybody, raising the feeling that we were not prepared and even mining the trust of many sectors of the population on the capability of science to overcome the crisis. Nonetheless, to say that what happened was not predictable and that we could not have done better would not only be inaccurate but also accessory by negligence.

In the history of humankind, we have never been so technologically capable of dealing with a pandemic, yet we have performed relatively poorly in many aspects. Setting aside the social, political, and economical nuances that have hindered the best-case scenario, as well as the intrinsic uncertainty associated with an event of this magnitude, the untimely commitment to long-known research necessities has been a decisive factor in the inadequate response.

In far too many cases, the trend has been to massively research ongoing threats, often fueled by politically driven funding, to later quit abruptly after the peril has passed or when the media interest has diminished. The research was thus rendered incomplete and did not yield any real solutions, even when results were very close. This behavior has left us in no significantly better condition to cope with the recurrence of similar situations despite the laudable efforts of scientists during times of crisis.

**Fig. 1.**
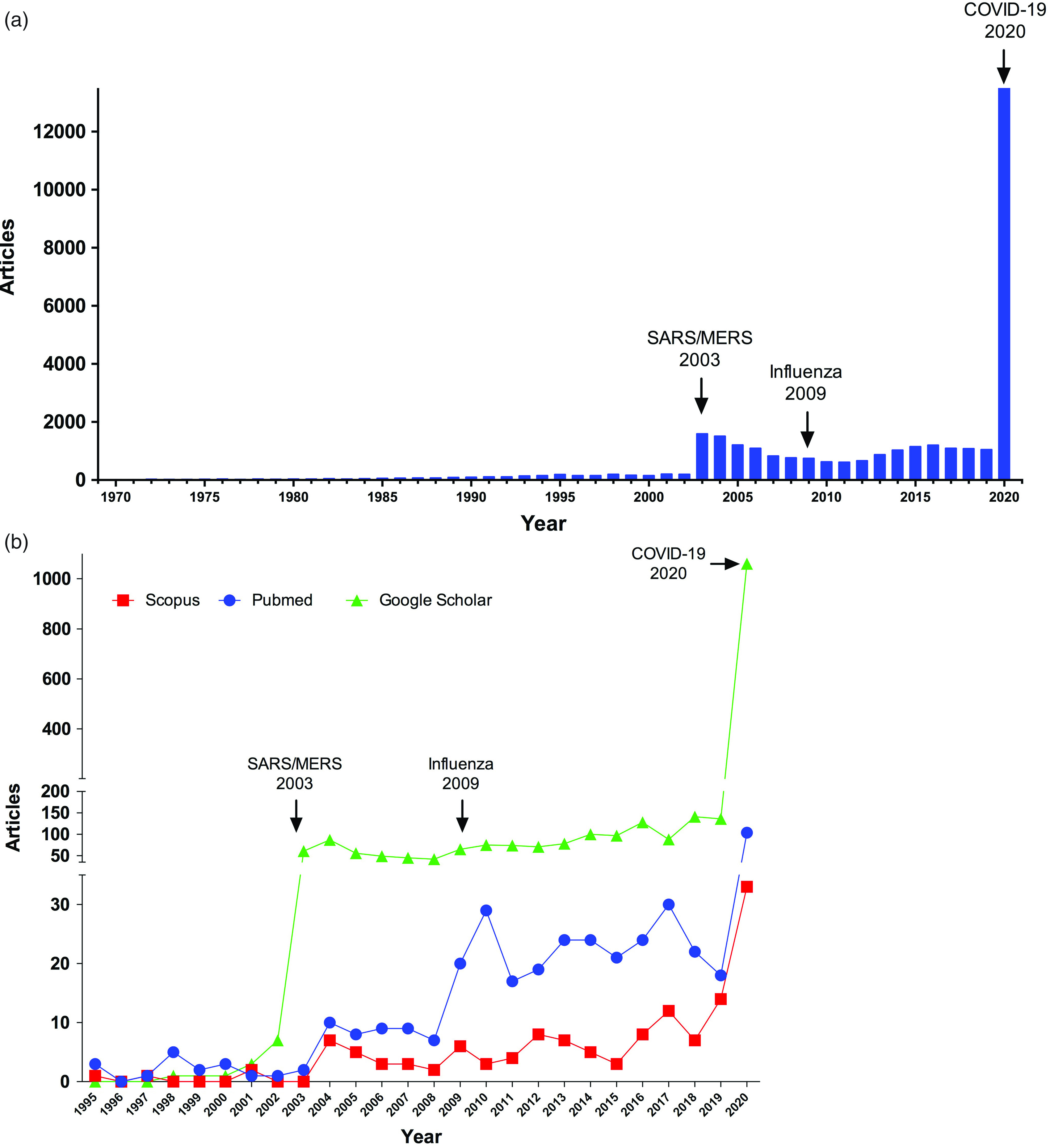
(a) Articles published containing the words SARS-CoV-2, SARS-CoV-1, COVID-19, SARS, or MERS. (b) Peaks of publications in 2003 and 2009 are evident, probably related to the outbreaks of SARS and influenza, respectively. The same pattern is observed in publications related to N95 masks from 1995 to 2020.

Over the past 50 years, research in PubMed (ie, using search terms, SARS-CoV-2 or SARS-CoV-1 or COVID-19 or SARS or MERS or coronavirus on June 15, 2020) regarding the Coronaviridae family has peaked during outbreaks and has flattened between them (Fig. 1a). This dynamic is also evident in research regarding technologies that are crucial for the management of epidemics, such as personal protective equipment (PPE). Medical masks and facepiece respirators, such as N95, are intended for single use. However, during outbreaks, shortages are inevitable and single use is overlooked. In recent decades, alternatives for reusable masks or safe disinfection protocols have been explored only in times of pressing need (Fig. 1b), and clearly, we were not well prepared in this aspect when COVID-19 broke out.

The severe acute respiratory syndrome (SARS) 2002–2004 epidemic could have served us better had we attended carefully to the lessons of that time. The evidence that presaged our current reality was recklessly and inexcusably overlooked. For more than a decade, the world disregarded evidence showing that wildlife markets in China, along with the high genetic recombination rates of coronaviruses, comprised an environment ripe for another zoonotic outbreak.^1^ We also missed a chance to achieve the only definitive solution to the pandemic when several promising SARS vaccines, which had undergone preclinical trials, were thwarted by a lack of further funding.^2^ Although SARS and SARS-CoV-2 are different viruses, their genetic closeness and similarity in the molecular mechanism of infection would have saved valuable time in the proper development of a SARS-CoV-2 vaccine. Instead, we are now rushing phase 1 clinical trials without preclinical or animal models.^2^

This pattern of scattered research might be a trademark of the way in which science has operated in contemporary society, but the vulnerability derived from allowing it to persist this way is unreasonable. Newton’s exceptionally hackneyed quote, “If I have seen further it is by standing on the shoulders of giants,” superbly conveys the notion of science being a cooperative effort, and we must always remember that these shoulders are often broadly spread across time. Public funding, as well as the overall mentality underlying research, cannot be steered toward achieving results in the short term or, otherwise, not achieving any results at all. Some processes, such as new PPE technologies and vaccines, must be understood and acknowledged as intrinsically time-consuming and must be continuously supported outside times of critical necessity. As evidence during the COVID-19 crisis shows, the real-time capacity to find solutions is insufficient and the price that we must pay for missed opportunities it is too high.
